# Supplementary material for: Patterns of mtDNA introgression suggest population replacement in Palaearctic whiskered bat species
Source: R Soc Open Sci. 2020 Jun 3;7(6):191805. doi: 10.1098/rsos.191805 (PMC7353987; doi:10.1098/rsos.191805)
Supplement: Supplementary Figures [file rsos191805supp1.pdf]

Supplementary Material

Patterns of mtDNA introgression suggest population replacement in Palearctic whiskered bat species

Emrah Çoraman[1, 2, 3, *, †], Heliana Dundarova[4, *], Christian Dietz[5], and Frieder Mayer[2, 6, †]

[1] Istanbul Technical University, Eurasia Institute of Earth Sciences, Department of Ecology and Evolution, Maslak, Istanbul, 34469, Turkey
[2] Museum für Naturkunde, Leibniz-Institut für Evolutions- und Biodiversitätsforschung, Berlin 10115, Germany
[3] Natural Science Collection, Martin-Luther-University Halle-Wittenberg, Domplatz 4, Halle (Saale) D-06108, Germany
[4] Bulgarian Academy of Sciences, Institute of Biodiversity and Ecosystem Research, 1 Tsar Osvoboditel, Sofia 1000, Bulgaria
[5] Biologische Gutachten Dietz, Balinger Str. 15, 72401 Haigerloch, Germany
[6] Berlin-Brandenburg Institute of Advanced Biodiversity Research (BBIB), Altensteinstraße 6, 14195 Berlin, Germany

[*] These authors contributed equally to this work.
[†] Corresponding author.

Supplementary Figures

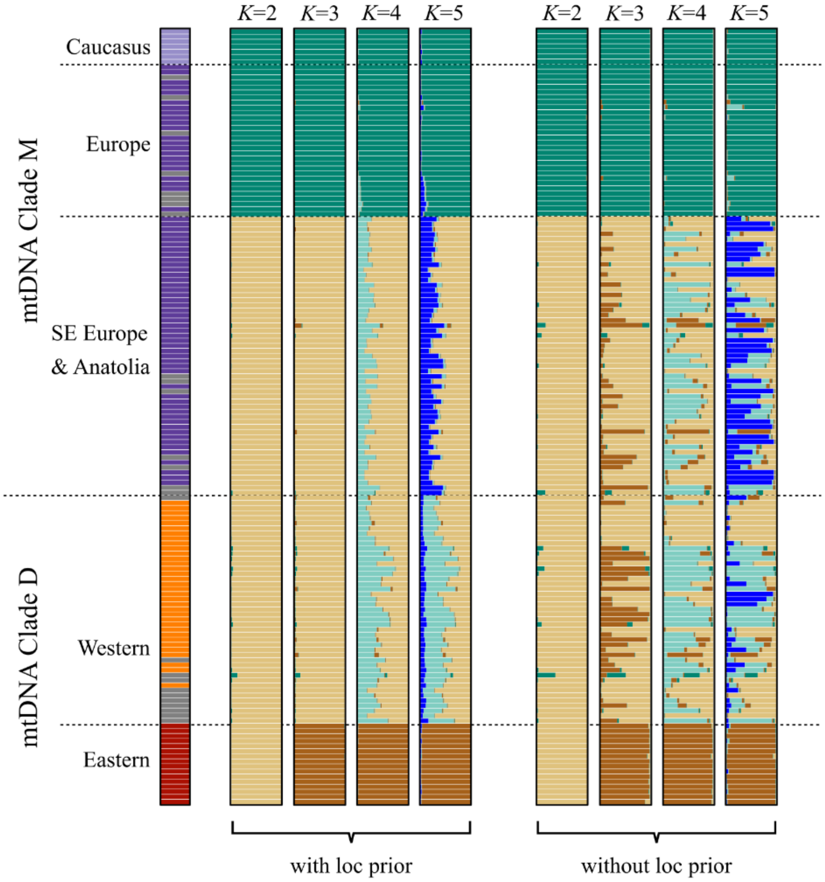

Supplementary figure 1. STRUCTURE results for *K*=2-5 with and without the loc prior model. The left panel shows the mtDNA assignments of the analysed samples. Individuals which did not have mtDNA sequences are colored as grey.

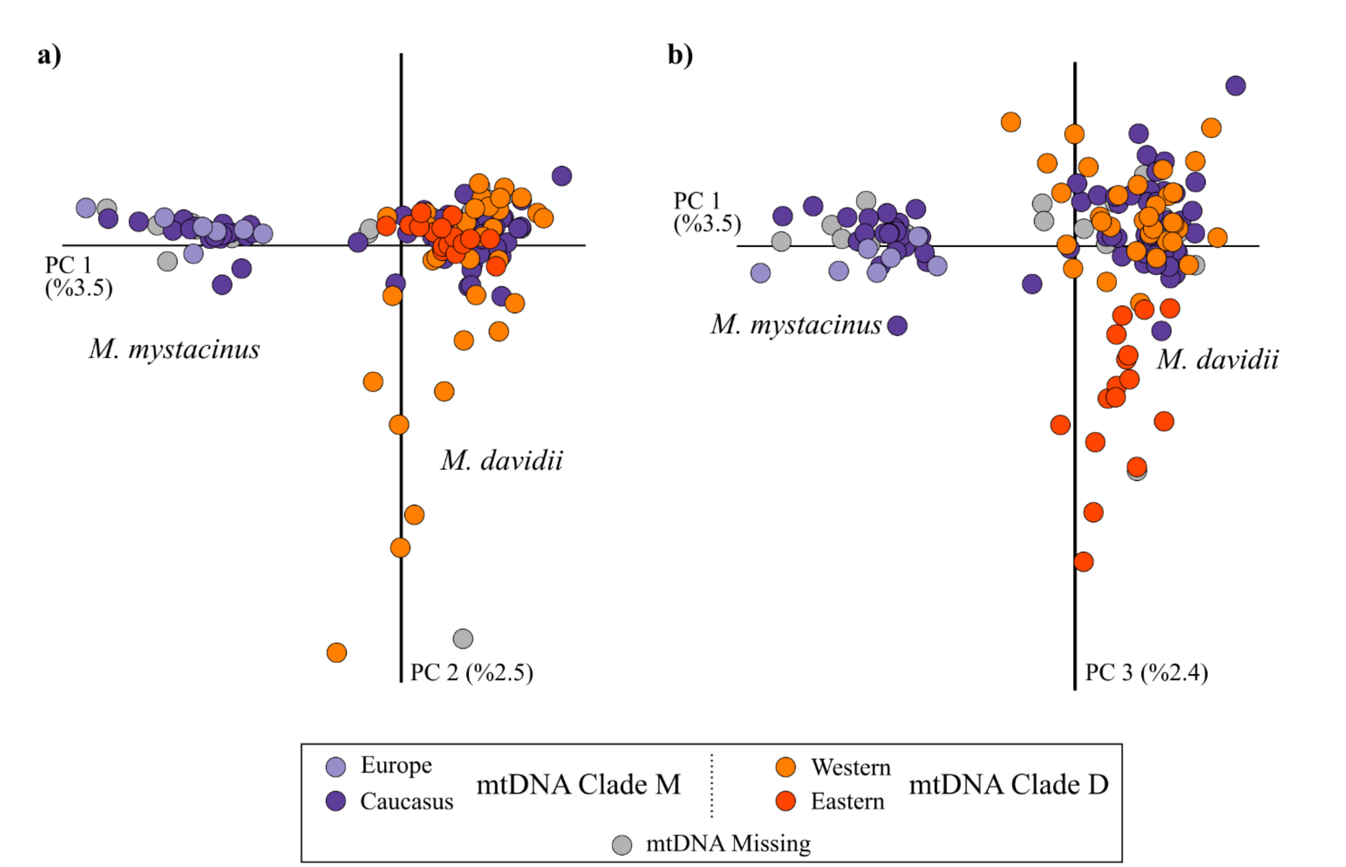

Supplementary figure 2. PCA; (a) PC1 vs. PC2, (b) PC1 vs. PC3. Circles are coloured based on the mtDNA assignments. Individuals which did not have mtDNA sequences are colored as grey.

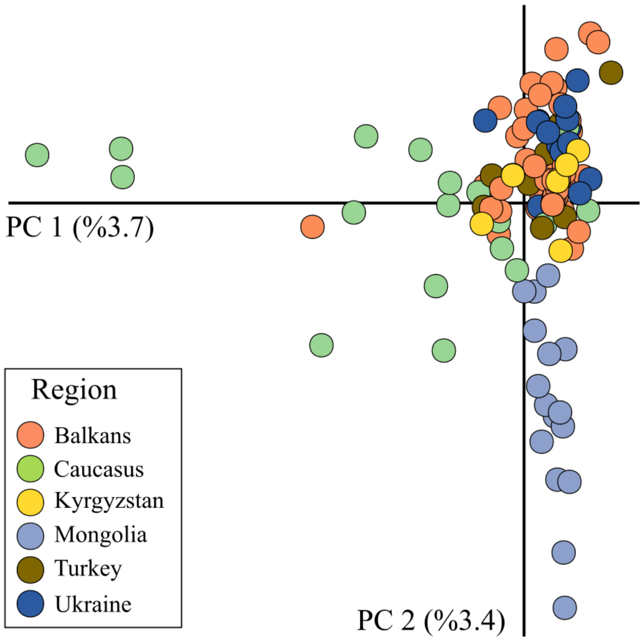

Supplementary figure 3. PCA for *M. davidii*.; circles are coloured based on the geographical regions.

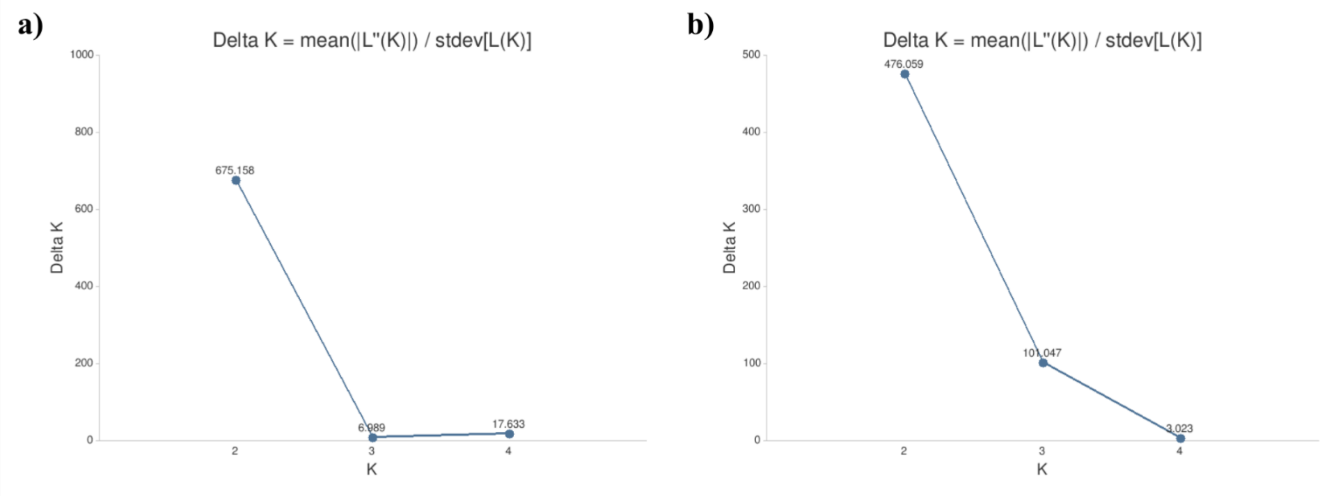

Supplementary figure 4. Optimal K by the Evanno method (Evanno et al., 2005) for the runs with (a) and without (b) the loc prior model.
