# Supplementary material for: Patterns of mtDNA introgression suggest population replacement in Palaearctic whiskered bat species
Source: R Soc Open Sci. 2020 Jun 3;7(6):191805. doi: 10.1098/rsos.191805 (PMC7353987; doi:10.1098/rsos.191805)
Supplement: Appendix [file rsos191805supp2.pdf]

Supplementary Material

Patterns of mtDNA introgression suggest population replacement in Palearctic whiskered bat species

Emrah Çoraman[1, 2, 3, *, †], Heliana Dundarova[4, *], Christian Dietz[5], and Frieder Mayer[2, 6, †]

[1] Istanbul Technical University, Eurasia Institute of Earth Sciences, Department of Ecology and Evolution, Maslak, Istanbul, 34469, Turkey

[2] Museum für Naturkunde, Leibniz-Institut für Evolutions- und Biodiversitätsforschung, Berlin 10115, Germany

[3] Natural Science Collection, Martin-Luther-University Halle-Wittenberg, Domplatz 4, Halle (Saale) D-06108, Germany

[4] Bulgarian Academy of Sciences, Institute of Biodiversity and Ecosystem Research, 1 Tsar Osvoboditel, Sofia 1000, Bulgaria

[5] Biologische Gutachten Dietz, Balinger Str. 15, 72401 Haigerloch, Germany

[6] Berlin-Brandenburg Institute of Advanced Biodiversity Research (BBIB), Altensteinstraße 6, 14195 Berlin, Germany

[*] These authors contributed equally to this work.

[†] Corresponding author.

Appendix 1. List of samples used in this study. Columns show: sample ID (ID); species, mitochondrial lineage membership (Mmys: *M. mystacinus*, Mdav: *M. davidii*, Mbra: *M. brandtii*); nuclear lineage membership (nuDNA); ISO 3166-1 alpha-3 country codes (Ctry); coordinates of the sampling locality, latitude (Lat) and longitude (Long); and the GenBank accession numbers of the sequenced genetic markers.

ID	Species	mtDNA	nuDNA	Ctry	Lat	Long	ND1	ABHD11	ACOX2	COPS	ROGDI_2
2298	*M. mystacinus*	Mmys	Mmys	MAR	35.57	-5.35	AY027840	MN706627	MN707021	-	MN706731
2314	*M. mystacinus*	Mmys	Mmys	HUN	47.53	21.64	AY027839	MN706628	MN707022	MN706884	MN706732
MspB4741	*M. davidii*	Mdav	Mdav	MNG	46.16	105.75	FR848521	MN706641	MN707037	-	MN706746
MspB4742	*M. davidii*	Mdav	Mdav	MNG	46.16	105.75	FR848522	MN706642	MN707038	-	MN706747
MspB4747	*M. davidii*	Mdav	Mdav	MNG	46.16	105.75	FR848527	MN706643	MN707039	-	MN706748
MspB4755	*M. davidii*	Mdav	Mdav	MNG	44.82	100.81	FR848529	-	MN707040	MN706896	MN706749
MspB4756	*M. davidii*	Mdav	Mdav	MNG	44.82	100.81	FR848530	MN706644	MN707041	-	MN706750
MspB4758	*M. davidii*	Mdav	Mdav	MNG	44.62	99.25	FR848532	MN706645	MN707042	-	MN706751
MspB4759	*M. davidii*	Mdav	Mdav	MNG	44.18	100.73	FR848533	MN706646	MN707043	MN706897	MN706752
MspB4760	*M. davidii*	Mdav	Mdav	MNG	46.18	100.66	FR848534	MN706647	MN707044	MN706898	MN706753
MspB4761	*M. davidii*	Mdav	Mdav	MNG	46.18	100.66	FR848535	MN706648	MN707045	-	MN706754
MspB4763	*M. davidii*	Mdav	Mdav	MNG	46.18	100.66	FR848536	MN706649	MN707046	MN706899	MN706755
MspB4764	*M. davidii*	Mdav	Mdav	MNG	47.26	105.47	FR848537	MN706650	MN707047	MN706900	MN706756
MspB4765	*M. davidii*	Mdav	Mdav	MNG	47.26	105.47	FR848530	MN706651	MN707048	-	MN706757
MspB4766	*M. davidii*	Mdav	Mdav	MNG	47.26	105.47	FR848539	MN706652	MN707049	-	MN706758
MspB5878	*M. davidii*	Mdav	Mdav	MNG	49.53	114.65	FR848516	MN706657	MN707055	-	MN706763
MspB6051	*M. davidii*	Mdav	Mdav	MNG	46.13	99.26	FR848541	MN706658	MN707056	MN706903	MN706764
MspB6057	*M. davidii*	Mdav	Mdav	MNG	46.13	99.26	FR848544	MN706659	MN707057	-	MN706765
ZMB_Mam_108331	*M. davidii*	Mmys	Mdav	TUR	38.49	28.37	MN707181	MN706629	MN707023	MN706885	MN706733
ZMB_Mam_108338	*M. davidii*	Mmys	Mdav	HRV	43.17	16.5	MN707188	MN706630	MN707024	-	MN706734
ZMB_Mam_108339	*M. davidii*	Mmys	Mdav	HRV	43.17	16.5	MN707189	MN706631	MN707025	MN706886	MN706735
ZMB_Mam_108340	*M. davidii*	Mmys	Mdav	HRV	43.17	16.5	MN707190	MN706632	MN707026	MN706887	MN706736
ZMB_Mam_108343	*M. davidii*	Mmys	Mdav	BGR	43.59	24.91	MN707193	MN706633	MN707027	-	MN706737
ZMB_Mam_108344	*M. davidii*	Mmys	Mdav	BGR	43.59	24.91	MN707194	MN706634	MN707028	MN706888	MN706738
ZMB_Mam_108345	*M. davidii*	Mmys	Mdav	BGR	43.59	24.91	MN707195	-	MN707029	MN706889	MN706739
ZMB_Mam_108350	*M. davidii*	Mdav	Mdav	BGR	43.59	24.91	MN707200	-	MN707032	MN706892	MN706741
ZMB_Mam_108351	*M. davidii*	Mmys	Mdav	BGR	43.59	24.91	MN707201	MN706636	MN707033	-	MN706742
ZMB_Mam_108352	*M. mystacinus*	Mmys	Mmys	DEU	48.43	8.59	MN707202	MN706638	MN707035	MN706894	MN706744
ZMB_Mam_108360	*M. davidii*	Mmys	Mdav	BGR	42.73	24.9	MN707210	MN706639	MN707036	MN706895	MN706745
ZMB_Mam_108372	*M. mystacinus*	Mmys	Mmys	DEU	49.77	11.3	MN707222	-	MN707050	MN706901	MN706759
ZMB_Mam_108373	*M. mystacinus*	Mmys	Mmys	DEU	49.77	11.3	MN707223	MN706653	MN707051	-	MN706760
ZMB_Mam_108387	*M. davidii*	Mmys	Mdav	SVN	45.83	13.84	MN707237	MN706655	MN707053	-	MN706761
ZMB_Mam_108388	*M. davidii*	Mmys	Mdav	SRB	45.25	19.87	MN707238	MN706656	MN707054	-	MN706762
ZMB_Mam_108390	*M. davidii*	Mmys	Mdav	SVN	45.54	13.85	MN707240	MN706661	MN707059	MN706905	MN706767
ZMB_Mam_108391	*M. davidii*	Mmys	Mdav	SVN	45.54	13.85	MN707241	MN706662	MN707060	MN706906	MN706768
ZMB_Mam_108392	*M. davidii*	Mdav	Mdav	UKR	47.5	37.93	MN707242	MN706663	MN707061	MN706907	MN706769
ZMB_Mam_108393	*M. davidii*	Mdav	Mdav	UKR	48.66	37.69	MN707243	MN706664	MN707062	MN706908	MN706770
ZMB_Mam_108394	*M. davidii*	Mdav	Mdav	UKR	48.66	37.69	MN707244	-	MN707063	MN706909	MN706771

ID	Species	mtDNA	nuDNA	Ctry	Lat	Long	ND1	ABHD11	ACOX2	COPS	ROGDI_2
ZMB_Mam_108395	*M. davidii*	Mdav	Mdav	UKR	47.45	35.1	MN707245	MN706665	MN707064	MN706910	MN706772
ZMB_Mam_108397	*M. davidii*	Mdav	Mdav	UKR	47.38	37.83	MN707247	MN706667	MN707066	MN706912	MN706773
ZMB_Mam_108398	*M. davidii*	Mdav	Mdav	UKR	47.38	37.83	MN707248	MN706668	MN707067	MN706913	MN706774
ZMB_Mam_108399	*M. davidii*	Mdav	Mdav	UKR	45.35	35.1	MN707249	MN706669	MN707070	-	MN706777
ZMB_Mam_108400	*M. davidii*	Mdav	Mdav	UKR	44.92	34.7	MN707250	MN706670	MN707071	-	MN706778
ZMB_Mam_108402	*M. davidii*	Mmys	Mdav	BGR	42.77	26.68	MN707252	-	MN707072	MN706916	MN706779
ZMB_Mam_108404	*M. davidii*	Mmys	Mdav	BGR	42.48	24.24	MN707254	-	MN707074	MN706918	MN706781
ZMB_Mam_108405	*M. mystacinus*	Mmys	Mmys	BGR	41.79	23.46	MN707255	-	MN707075	MN706919	MN706782
ZMB_Mam_108409	*M. davidii*	Mmys	Mdav	GRC	40.09	20.93	MN707259	MN706671	MN707076	-	MN706783
ZMB_Mam_108420	*M. davidii*	Mmys	Mdav	BGR	43.69	24.89	MN707270	-	MN707077	MN706920	MN706784
ZMB_Mam_108421	*M. davidii*	Mmys	Mdav	BGR	43.69	24.89	MN707271	-	MN707078	MN706921	MN706785
ZMB_Mam_108422	*M. davidii*	Mmys	Mdav	BGR	43.63	24.85	MN707272	-	MN707079	MN706922	MN706786
ZMB_Mam_108425	*M. davidii*	Mmys	Mdav	BGR	43.59	24.91	MN707275	-	MN707082	MN706925	MN706787
ZMB_Mam_108426	*M. davidii*	Mdav	Mdav	BGR	43.59	24.91	MN707276	MN706672	MN707083	MN706926	MN706788
ZMB_Mam_108428	*M. davidii*	Mmys	Mdav	BGR	43.59	24.91	MN707278	-	MN707084	MN706927	MN706789
ZMB_Mam_108429	*M. davidii*	Mmys	Mdav	BGR	43.59	24.91	MN707279	-	MN707087	MN706930	MN706792
ZMB_Mam_108430	*M. davidii*	Mmys	Mdav	BGR	43.69	24.89	MN707280	-	MN707089	MN706932	MN706794
ZMB_Mam_108431	*M. davidii*	Mmys	Mdav	BGR	43.69	24.89	MN707281	-	MN707090	MN706933	MN706795
ZMB_Mam_108432	*M. davidii*	Mmys	Mdav	BGR	43.69	24.89	MN707282	-	MN707091	MN706934	MN706796
ZMB_Mam_108433	*M. davidii*	Mmys	Mdav	BGR	43.69	24.89	MN707283	-	MN707092	MN706935	MN706797
ZMB_Mam_108434	*M. davidii*	Mmys	Mdav	BGR	43.63	24.85	MN707284	-	MN707093	MN706936	MN706798
ZMB_Mam_108437	*M. davidii*	Mmys	Mdav	BGR	43.63	24.85	MN707285	-	MN707094	MN706937	MN706799
ZMB_Mam_108440	*M. davidii*	Mdav	Mdav	ARM	40.42	45.45	MN707288	MN706673	MN707095	-	MN706800
ZMB_Mam_108441	*M. davidii*	Mdav	Mdav	ARM	40.42	45.45	MN707289	MN706674	MN707096	MN706938	MN706801
ZMB_Mam_108442	*M. davidii*	Mdav	Mdav	ARM	40.42	45.45	MN707290	MN706675	MN707097	MN706939	MN706802
ZMB_Mam_108443	*M. davidii*	Mdav	Mdav	ARM	40.42	45.45	MN707291	MN706676	MN707098	MN706940	MN706803
ZMB_Mam_108444	*M. davidii*	Mdav	Mdav	ARM	40.42	45.45	MN707292	MN706677	MN707099	MN706941	MN706804
ZMB_Mam_108445	*M. davidii*	Mdav	Mdav	ARM	40.42	45.45	MN707293	MN706678	MN707100	MN706942	MN706805
ZMB_Mam_108448	*M. davidii*	Mdav	Mdav	ARM	40.42	45.45	MN707296	MN706679	MN707101	MN706943	MN706806
ZMB_Mam_108449	*M. davidii*	Mdav	Mdav	ARM	40.42	45.45	MN707297	MN706680	MN707102	MN706944	MN706807
ZMB_Mam_108454	*M. davidii*	Mdav	Mdav	ARM	39.39	46.26	MN707302	MN706681	MN707103	MN706945	MN706808
ZMB_Mam_108455	*M. davidii*	Mdav	Mdav	ARM	39.68	45.24	MN707303	MN706682	MN707104	-	MN706809
ZMB_Mam_108456	*M. davidii*	Mdav	Mdav	ARM	39.68	45.24	MN707304	-	MN707105	MN706946	MN706810
ZMB_Mam_108458	*M. mystacinus*	Mmys	Mmys	ROU	46.33	22.29	MN707306	MN706684	MN707107	MN706948	MN706811
ZMB_Mam_108461	*M. mystacinus*	Mmys	Mmys	GEO	41.53	44.15	MN707309	MN706686	MN707109	MN706950	MN706812
ZMB_Mam_108462	*M. davidii*	Mdav	Mdav	GEO	41.46	44.24	MN707310	MN706687	MN707110	MN706951	MN706813
ZMB_Mam_108463	*M. davidii*	Mdav	Mdav	GEO	41.78	44.64	MN707311	MN706688	MN707111	-	MN706814
ZMB_Mam_108464	*M. mystacinus*	Mmys	Mmys	GEO	41.91	46.07	MN707312	MN706689	MN707112	MN706952	MN706815
ZMB_Mam_108465	*M. mystacinus*	Mmys	Mmys	GEO	41.8	45.19	MN707313	MN706690	MN707113	MN706953	MN706816
ZMB_Mam_108466	*M. mystacinus*	Mmys	Mmys	GEO	41.84	46.29	MN707314	MN706691	MN707114	MN706954	MN706817
ZMB_Mam_108468	*M. davidii*	Mdav	Mdav	GEO	41.59	44.49	MN707316	MN706692	MN707115	-	MN706818
ZMB_Mam_108469	*M. davidii*	Mdav	Mdav	GEO	41.59	44.49	MN707317	MN706693	MN707116	-	MN706819
ZMB_Mam_108478	*M. davidii*	Mdav	Mdav	GEO	41.49	44.46	MN707326	MN706694	MN707117	MN706955	MN706820
ZMB_Mam_108479	*M. davidii*	Mdav	Mdav	GEO	41.49	44.46	MN707327	MN706695	MN707118	MN706956	MN706821
ZMB_Mam_108480	*M. mystacinus*	Mmys	Mmys	GEO	41.85	46.4	MN707328	MN706696	MN707119	MN706957	MN706822
ZMB_Mam_108481	*M. mystacinus*	Mmys	Mmys	RUS	54.74	40.95	MN707330	MN706698	MN707121	MN706959	MN706824
ZMB_Mam_108482	*M. davidii*	Mmys	Mdav	ALB	42	19.63	MN707331	-	MN707122	MN706960	MN706825
ZMB_Mam_108483	*M. davidii*	Mmys	Mdav	ALB	42	19.63	MN707332	-	MN707123	MN706961	MN706826
ZMB_Mam_108486	*M. davidii*	Mmys	Mdav	ALB	41.33	20.1	MN707335	-	MN707124	MN706962	MN706827
ZMB_Mam_108487	*M. davidii*	Mmys	Mdav	ALB	42.06	19.53	MN707336	-	MN707125	MN706963	MN706828
ZMB_Mam_108490	*M. mystacinus*	Mmys	Mmys	POL	50.82	15.82	MN707339	MN706701	MN707128	-	MN706829
ZMB_Mam_108491	*M. mystacinus*	Mmys	Mmys	POL	49.14	22.57	MN707340	MN706703	MN707130	MN706967	MN706830
ZMB_Mam_108494	*M. mystacinus*	Mmys	Mmys	POL	49.48	22.66	MN707343	MN706706	-	MN706970	MN706832
ZMB_Mam_108495	*M. davidii*	Mmys	Mdav	GRC	38.31	20.48	MN707344	-	MN707132	MN706971	MN706833
ZMB_Mam_108496	*M. davidii*	Mmys	Mdav	GRC	38.31	20.48	MN707345	-	MN707133	MN706972	MN706834
ZMB_Mam_108497	*M. davidii*	Mmys	Mdav	TUR	40.9	33.65	MN707346	MN706707	MN707134	MN706973	MN706835
ZMB_Mam_108498	*M. davidii*	Mmys	Mdav	TUR	40.9	33.65	MN707347	MN706708	-	MN706974	MN706836
ZMB_Mam_108499	*M. davidii*	Mmys	Mdav	TUR	40.72	34.72	MN707348	MN706709	MN707135	MN706975	MN706837
ZMB_Mam_108500	*M. davidii*	Mmys	Mdav	TUR	40.72	34.72	MN707349	MN706710	MN707136	MN706976	MN706838
ZMB_Mam_108501	*M. mystacinus*	Mmys	Mmys	TUR	40.72	34.72	MN707350	MN706711	MN707137	MN706977	MN706839
ZMB_Mam_108502	*M. davidii*	Mmys	Mdav	TUR	40.72	34.72	MN707351	MN706712	-	MN706978	MN706840
ZMB_Mam_108504	*M. davidii*	Mmys	Mdav	TUR	37.26	31.76	MN707353	MN706714	MN707139	MN706980	MN706841
ZMB_Mam_108505	*M. davidii*	Mmys	Mdav	TUR	38.74	29.75	MN707354	MN706715	-	MN706981	MN706842
ZMB_Mam_108506	*M. davidii*	Mmys	Mdav	GRC	35.11	24.87	MN707355	-	MN707141	MN706984	MN706844
ZMB_Mam_108508	*M. mystacinus*	Mmys	Mmys	DEU	49.73	11.08	MN707357	MN706716	-	MN706987	MN706848
ZMB_Mam_108511	*M. mystacinus*	Mmys	Mmys	BGR	41.65	24.53	MN707360	-	MN707145	MN706989	MN706849
ZMB_Mam_108512	*M. mystacinus*	Mmys	Mmys	BGR	41.65	24.53	MN707361	-	MN707146	MN706990	MN706850
ZMB_Mam_108520	*M. davidii*	Mmys	Mdav	HRV	44.28	15.31	MN707369	-	MN707148	MN706992	MN706852
ZMB_Mam_108521	*M. davidii*	Mmys	Mdav	HRV	44.28	15.31	MN707370	-	MN707149	MN706993	MN706853
ZMB_Mam_108525	*M.* cf. *davidii*	Mmys	-	HRV	44.35	15.48	MN707374	-	-	MN706994	MN706854
ZMB_Mam_108526	*M. davidii*	Mmys	Mdav	HRV	44.28	15.31	MN707375	-	MN707150	MN706995	MN706855
ZMB_Mam_108531	*M. mystacinus*	Mmys	Mmys	BGR	41.54	23.65	MN707380	-	MN707153	MN706999	MN706859
ZMB_Mam_108535	*M.* cf. *mystacinus*	Mmys	-	ISR	33.31	35.77	MN707384	-	-	-	MN706863
ZMB_Mam_108536	*M.* cf. *mystacinus*	Mmys	-	ISR	33.31	35.77	MN707385	-	-	MN707003	MN706864
ZMB_Mam_108538	*M.* cf. *mystacinus*	Mmys	-	ISR	33.31	35.77	MN707387	-	MN707157	-	MN706865
ZMB_Mam_108541	*M.* cf. *mystacinus*	Mmys	-	ISR	33.31	35.77	MN707390	-	-	-	MN706875
ZMB_Mam_108557	*M. davidii*	Mmys	Mdav	ARM	39.09	46.47	MN707406	MN706721	MN707168	-	MN706876

ID	Species	mtDNA	nuDNA	Ctry	Lat	Long	ND1	ABHD11	ACOX2	COPS	ROGDI_2
ZMB_Mam_108561	*M. davidii*	-	Mdav	KGZ	41.31	72.96	-	MN706723	MN707171	MN707013	MN706877
ZMB_Mam_108562	*M. davidii*	Mdav	Mdav	KGZ	41.31	72.96	MN707410	MN706724	MN707172	MN707014	MN706878
ZMB_Mam_108563	*M. davidii*	Mdav	Mdav	KGZ	41.31	72.96	MN707411	MN706725	MN707173	MN707015	MN706879
ZMB_Mam_108564	*M. davidii*	Mdav	Mdav	KGZ	41.31	72.96	MN707412	MN706726	MN707174	MN707016	MN706880
ZMB_Mam_108566	*M. davidii*	-	Mdav	KGZ	41.31	72.96	-	MN706728	MN707176	MN707018	MN706881
ZMB_Mam_108567	*M. davidii*	Mmys	Mdav	SVN	46.26	14.02	MN707414	MN706729	MN707177	MN707019	MN706882
ZMB_Mam_108568	*M. mystacinus*	Mmys	Mmys	AUT	47.79	12.99	MN707415	MN706730	-	MN707020	MN706883
ZMB_Mam_108571	*M. davidii*	-	Mdav	BGR	43.59	24.91	-	MN706635	MN707031	MN706891	MN706740
ZMB_Mam_108572	*M. mystacinus*	-	Mmys	DEU	48.43	8.59	-	MN706637	MN707034	MN706893	MN706743
ZMB_Mam_108573	*M. mystacinus*	-	Mmys	SVN	45.95	15.16	-	MN706660	MN707058	MN706904	MN706766
ZMB_Mam_108574	*M. davidii*	-	Mdav	UKR	47.38	37.83	-	-	MN707068	MN706914	MN706775
ZMB_Mam_108575	*M. davidii*	-	Mdav	UKR	47.38	37.83	-	-	MN707069	MN706915	MN706776
ZMB_Mam_108576	*M. mystacinus*	-	Mmys	BGR	41.76	23.54	-	-	MN707073	MN706917	MN706780
ZMB_Mam_108577	*M. davidii*	-	Mdav	BGR	43.59	24.91	-	-	MN707085	MN706928	MN706790
ZMB_Mam_108578	*M. davidii*	-	Mdav	BGR	43.59	24.91	-	-	MN707086	MN706929	MN706791
ZMB_Mam_108579	*M. davidii*	-	Mdav	BGR	43.59	24.91	-	-	MN707088	MN706931	MN706793
ZMB_Mam_108581	*M. mystacinus*	-	-	POL	49.2	22.67	-	-	-	-	MN706831
ZMB_Mam_108583	*M. davidii*	-	Mdav	GRC	35.31	23.94	-	-	MN707140	MN706983	MN706843
ZMB_Mam_108584	*M.* cf. *davidii*	-	-	GRC	35.59	24.15	-	-	-	MN706985	MN706845
ZMB_Mam_108585	*M. davidii*	-	Mdav	GRC	35.59	24.15	-	-	MN707142	MN706986	MN706846
ZMB_Mam_108586	*M. cf. davidii*	-	-	GRC	35.59	24.15	-	-	MN707143	-	MN706847
ZMB_Mam_108587	*M. mystacinus*	-	Mmys	SRB	43.31	19.81	-	-	MN707147	MN706991	MN706851
ZMB_Mam_108588	*M.* cf. *mystacinus*	-	-	BGR	41.65	23.37	-	-	-	MN706996	MN706856
ZMB_Mam_108589	*M. mystacinus*	-	Mmys	BGR	41.65	23.37	-	-	MN707151	MN706997	MN706857
ZMB_Mam_108590	*M. mystacinus*	-	Mmys	BGR	41.54	23.65	-	-	MN707152	MN706998	MN706858
ZMB_Mam_108591	*M. mystacinus*	-	Mmys	BGR	41.54	23.65	-	-	MN707154	MN707000	MN706860
ZMB_Mam_108592	*M.* cf. *mystacinus*	-	-	BGR	41.73	25.22	-	-	-	MN707001	MN706861
ZMB_Mam_108593	*M. davidii*	-	Mdav	BGR	41.65	24.53	-	-	MN707155	MN707002	MN706862
ZMB_Mam_108594	*M. davidii*	-	Mdav	BGR	43.25	22.96	-	-	MN707158	MN707004	MN706866
ZMB_Mam_108595	*M. davidii*	-	-	UKR	48.05	29.58	-	-	MN707159	-	MN706867
ZMB_Mam_108596	*M. davidii*	-	Mdav	UKR	47.43	33.92	-	-	MN707160	MN707005	MN706868
ZMB_Mam_108597	*M. davidii*	-	Mdav	UKR	47.43	33.92	-	-	MN707161	MN707006	MN706869
ZMB_Mam_108598	*M. davidii*	-	Mdav	UKR	47.43	33.92	-	-	MN707162	MN707007	MN706870
ZMB_Mam_108599	*M. davidii*	-	Mdav	UKR	46.71	30.61	-	-	MN707163	MN707008	MN706871
ZMB_Mam_108600	*M. davidii*	-	Mdav	UKR	46.71	30.61	-	-	MN707164	MN707009	MN706872
ZMB_Mam_108601	*M. davidii*	-	Mdav	UKR	46.71	30.61	-	-	MN707165	MN707010	MN706873
ZMB_Mam_108602	*M. davidii*	-	Mdav	UKR	46.71	30.61	-	-	MN707166	MN707011	MN706874
ZMB_Mam_108604	*M. brandtii*	Mbra	Mbra	Geo	41.89	46.21	MN707329	MN706697	MN707120	MN706958	MN706823

Appendix 2. List of the GenBank sequences used in this analysis.

AB106572, AB106573, AB106574, AB106575, AB106576, AB106577, AB106578, AB106579, AB106580, AB106581, AF401438, AY027836, AY027838, AY027839, AY027840, AY027842, AY027843, AY027844, AY027846, AY027847, AY027848, AY027849, AY027850, AY552330, AY552331, AY699856, AY699857, AY699858, AY699859, AY699860, AY699861, AY699862, AY699863, AY699865, AY699866, AY699867, DQ915033, FR848510, FR848511, FR848512, FR848513, FR848514, FR848515, FR848516, FR848518, FR848521, FR848522, FR848523, FR848526, FR848527, FR848528, FR848529, FR848530, FR848530_2, FR848532, FR848533, FR848534, FR848535, FR848536, FR848537, FR848539, FR848540, FR848541, FR848542, FR848543, FR848544, FR848545, FR848546, HQ529599, HQ529600, HQ529601, HQ529602, HQ529603, HQ529604, HQ529605, HQ529606, HQ529607, HQ529608, HQ529609, JX645262, JX645288, JX645289, JX645290, JX645291, JX645292, JX645293, JX645294, JX645295, JX645296, JX645297, JX645298, JX645299, JX645300, JX645301, JX645302, JX645303, JX645304, JX645305, JX645306, JX645307, JX645308, JX645309, JX645310, JX645311, JX645312, JX645313, JX645314, JX645315, JX645316, JX645317, JX645318, JX645319, KF111724, KF218452, KF218453, KF218475, KF218476, KF218477, KF218478, KF218479, KF218480, KF218481, KF218482, KJ948240, KJ948271, KJ948272, KJ948273, KJ948274, KJ948275, KJ948276, KJ948277, KJ948278, KJ948279, KJ948280, KJ948281, KJ948282, KJ948283, KJ948284
